# Does the format of the adult ADHD self-report scale influence screen-positive rates? A randomized controlled trial in primary care

**DOI:** 10.3389/fpsyt.2025.1646293

**Published:** 2026-01-02

**Authors:** Roni Y. Kraut, Christian Ono, Scott Garrison, Omar Kamal, Marissa L. Doroshuk, Ben Vandermeer, Gerard Amanna, Oksana Babenko

**Affiliations:** 1 Department of Family Medicine, Faculty of Medicine & Dentistry, University of Alberta, Edmonton, AB, Canada; 2 Department of Family Medicine, Faculty of Health Sciences, Queen’s University, Kingston, ON, Canada; 3 Faculty of Medicine & Dentistry, Western University, London, ON, Canada; 4 Faculty of Engineering, University of Alberta, Edmonton, AB, Canada; 5 Epidemiology Coordinating and Research Centre, Faculty of Medicine & Dentistry, University of Alberta, Edmonton, AB, Canada

**Keywords:** ADHD, ASRS, malingering, screening, predictive value of test, adults, overdiagnosis, randomized controlled trial

## Abstract

**Objective:**

The Adult Attention-Deficit-Hyperactivity-Disorder (ADHD) Self-Report Scale (ASRS) is widely used for ADHD screening in primary care clinics worldwide. While it offers a quick and efficient method to screen for ADHD, it also has a high false positive rate. The standard ASRS format—shaded responses for screen-positive items and grouping the key questions in Part A—may contribute to this issue. The objective of this study is to examine whether these design features impact the screen positive rate.

**Methods:**

This is a 2x2 factorial randomized controlled trial that ran from July to October 2024. Individuals 19 to 65 years old attending a family medicine clinic received at random one of four ASRS forms on registration (standard, grouping only, shading only, and no shading and no grouping). Logistic regression was used to analyze the results.

**Results:**

A total of 595 participants completed the study: mean age 39 (standard deviation 12), 79% women, 85% with at least some post-secondary education, 54% White, 33% Asian, 13% other ethnicities. Additionally, 14% had a prior ADHD diagnosis, and 23% suspected they had undiagnosed ADHD. Overall, 32% of participants screened positive for ADHD, and grouping and shading were not statistically significant predictors of a positive ADHD screen (Odds ratio [OR] 1.25, 95% CI 0.98–1.58 and OR 0.88 95% CI 0.69–1.12 respectively). In contrast, prior ADHD diagnosis and suspected undiagnosed ADHD were statistically significant predictors (OR 47.4, 95% CI 23,1–97.0 and OR 16.2, 95% CI 9.7–27.2, respectively).

**Conclusion:**

The standard ASRS does not appear to increase the screen positive rate. Nevertheless, the high positive screening rate highlights the need for more effective ADHD screening tools in primary care.

**Clinical trial registration:**

Clinicaltrials.gov, identifier NCT06530758.

## Introduction

Attention deficit hyperactivity disorder (ADHD) is a chronic condition affecting an estimated 2%–7% of the adult population, and is associated with significant impairments in behavioral, cognitive, and social functioning (1–5). Limited access to specialized medical professionals for ADHD assessment and management worldwide has raised concerns about underdiagnosis and undertreatment, and screening tools have been developed to facilitate diagnosis in primary care (6–8).

One such screening tool is the Adult ADHD Self-Report Scale (ASRS) that was developed in 2005 in collaboration with the World Health Organization (8). The ASRS is now one of the most widely used ADHD screening tools for adults in primary care due to its brevity, simplicity, and comprehensiveness (9, 10). The ASRS has a high negative predictive value (+95%), meaning a negative screen strongly suggests the absence of ADHD (11). However, its positive predictive value is relatively low, estimated between 12%–22%, suggesting that a positive screen result is more likely to be a false alarm, with only a minority of individuals who screen positive actually having ADHD (11, 12).

The ASRS consists of 18 questions aligned with DSM-IV criteria, with responses considered indicative of ADHD symptoms (i.e., qualifying responses) ranging from *“sometimes”* to *“often”* depending on the question (8). To fit within the time constraints of primary care, the ASRS was revised to divide questions into Part A, comprising the six most sensitive and specific items, and Part B, containing the remaining 12 items for additional context (13). A positive screen is typically defined as at least 4 of the 6 Part A items meeting their respective thresholds (13). For ease of scoring, the standard ASRS format *shades* the qualifying response options and *groups* Part A and Part B items under separate headings (14).

Substantial research on survey design has found that the layout (e.g., question grouping) and visual design (e.g., color) of a survey can influence the validity and reliability of its results. For example, satisficing, when respondents choose an answer that is acceptable rather than optimal, tends to increase when similar questions are grouped together (15). Additionally, the use of different color hues between response options has been found to increase the selection of more extreme responses, especially when response options do not have a label (16).

The aim of this study was to evaluate whether these design features of the ASRS—shading and question grouping—influence the rate of positive screening results. Our hypothesis was that removing these features may lower the positive screen rate and potentially improve the positive predictive value of the ASRS.

## Methods

This is a 2x2 factorial design randomized controlled trial. The trial was registered on clincaltrials.gov July 30, 2024 (https://clinicaltrials.gov/study/NCT06530758). It was approved by the University of Alberta Health Research Ethics Board (Pro00144202). This trial is reported using the CONSORT 2025 reporting guidelines. (17).

### Setting

The study was conducted at Shifa Medical Clinic, a primary care clinic in Edmonton, Canada from July – October 2024. The clinic has 12 physicians and sees around 2000 patients per month.

### Participant eligibility criteria

All patients within the age ranges of 19 to 65 years who checked in for an appointment at Shifa Medical Clinic and were expected to be in the waiting room for at least 5 minutes before their appointment were eligible. Patients were excluded if they were unable to complete the survey, which included those with cognitive impairments, difficulty using a computer, or limited English literacy.

### Survey

The survey had questions on demographics and ADHD status (diagnosed vs suspected ADHD) and included one of the four possible ASRS versions (**Table 1**, **Supplementary Materials 1, 2**). To implement this, a website was designed with JavaScript functionality, and a Google Form was set up with the same questions as the website. Responses were automatically transferred to Google Form, which then populated a Google Sheet for analysis. A basic JavaScript randomization function (using a uniform distribution with a 25% chance for each version) assigned one of the four ASRS versions to participants. To verify that the randomization function operated correctly, the function was run over 20,000 times, yielding output consistent with a uniform distribution. The JavaScript and HTML code are available at: https://github.com/VINXIS/kraut-adhd-form.

**Table 1:** ASRS Versions.

	Shaded	Not Shaded
Grouped	Standard	Grouping only
Not grouped	Shading only	Neither

Shaded - Positive answers are indicated with a shaded checkbox.

Not shaded - Positive answers do not have a shaded checkbox.

Grouped - Parts A and B questions are separated by headings.

Not Grouped - Parts A and B questions are not separated by headings.

### Patient involvement

The survey was piloted with approximately 20 patients prior to launching to ensure that it was easy to complete, clearly understandable, and would not disrupt clinic workflow (< 5 minutes to complete). The original survey included the ADHD Symptoms Infrequency Scale (ASIS) to detect malingering (18); this was excluded in the final survey, as during the survey pilot, patients felt it was too lengthy to complete, and the time required interrupted the clinic workflow.

### Clinic process

Shifa reception staff, that included 10 full- and part-time staff members, initiated the survey on a clinic laptop for eligible participants during check-in. There was full allocation concealment. Although staff knew the study involved different ASRS versions, they were unaware of the specific differences. Additionally, the survey opened to an implied consent form, obscuring the assigned ASRS version. While staff could theoretically click through to identify and eventually select a specific ASRS version, this would require repeated attempts due to randomization, and highly unlikely given the fast-paced, multitasking nature of reception work.

If a participant was ineligible or declined, the clinic staff selected the “no response” option on the first page and recorded the participant’s date of birth and sex from the electronic medical record and reason for no response: declined, already completed, unable to complete. During busy periods, staff were at times not able to offer the survey to patients. To capture this, the clinic administrator periodically compared the survey responses to the patients who attended the clinic on the same day using the clinic’s electronic medical record system, and then recorded the age and sex of patients without a survey response onto the Google drive spreadsheet.

### Blinding

Participants were fully blinded; while they were aware they would be receiving one of the versions of the ASRS, they were unaware whether it was one of the intervention versions (grouping only, shading only, neither) or the standard version. Clinic physicians were blinded and were usually unaware that the patient even completed the survey, given it was completed in the reception area. The study team and data analyst were not blinded.

### Outcome

The primary outcome of this trial was the screen-positive rate on the ASRS, measured as the percentage of participants who screened positive among all those who completed the survey. A screen-positive result was determined using a commonly applied scoring method for the ASRS, specifically, at least 4 out of 6 positive responses on Part A (13). Other scoring methods are included in the additional analysis (10).

### Sample size

Sample size was calculated using the z-test for proportions and based on the assumption of a 10% screen positive rate. A sample size of 1500 participants was needed to detect a difference of ≥3% of screen positive rates between survey versions with 80% power and an alpha of 0.05. At the approximate midpoint of the trial, the observed overall screen positive rate was much higher (30%) than expected; this allowed for a sample size of 600 rather than 1500 to achieve a relative difference equivalent of 3% for the higher observed screen positive rate of 30% (i.e., 9%).

### Statistical analysis

We used the chi-squared test for categorical variables and ANOVA for continuous variables to assess statistically significant differences in participant characteristics across the different ASRS versions. For any incomplete demographic data, participants were excluded from both the numerator and denominator for the specific variable in question. We also compared gender/sex (chi-squared) and age (ANOVA) between participants who completed the survey to those who declined and were not offered the survey. The gender/sex comparison was considered reasonable given the small number identifying as “other.”

We calculated the screen-positive rate for all participants and within subgroups defined as diagnosed, suspected, or neither. Participants who answered ‘yes’ to having received an ADHD diagnosis (survey question 5) were classified as diagnosed. Those who responded ‘likely’ or ‘very likely’ to the question about whether they believe they may have ADHD (survey question 5e) were categorized as suspecting to have ADHD.

Logistic regression was performed to determine the odds of a participant obtaining a positive ADHD screen among the different versions and included the following covariates: shading, grouping, shading x grouping, ADHD status, self-reported gender, and age. Age was analyzed as a continuous variable rather than a categorical variable based on previous findings stating that an inverse linear relationship exists between ADHD diagnoses and age (3). Irrespective of the version, if any of the variables were not completed in the survey (gender, age, ADHD status) or the survey was not complete (Part A or Part A and B depending on the scoring method), the participant was excluded from this analysis. In addition, we excluded participants that listed their gender as other (i.e., non-binary, transgender, other) from the regression, since this added complexity to the model without impacting the results.

We conducted several additional analyses, including: descriptive statistics on the characteristics of the participants diagnosed with ADHD; an assessment of whether there was any statistically significant differences in the proportion of participants offered the survey across days of the week using the Kruskal-Wallis test; and calculation of the total percentage of participants offered the survey in the morning compared to the afternoon.

Finally, we computed the screen positive percentage and performed logistic regression for 3 other scoring methods for the ASRS: 1) Part A and B, Dichotomous scoring (i.e., each question is scored as either positive or negative) with a cutoff of 9 for a positive screen; 2) Part A, Scaled scoring (i.e., 0 being never and 4 being very often) with a cutoff of 14 out of 24 for a positive screen; and 3) Part A and B, Scaled scoring (i.e., 0 being never and 4 being very often) with a cutoff of 35 out of 72 for a positive screen (10, 19).

## Results

**Figure 1:**
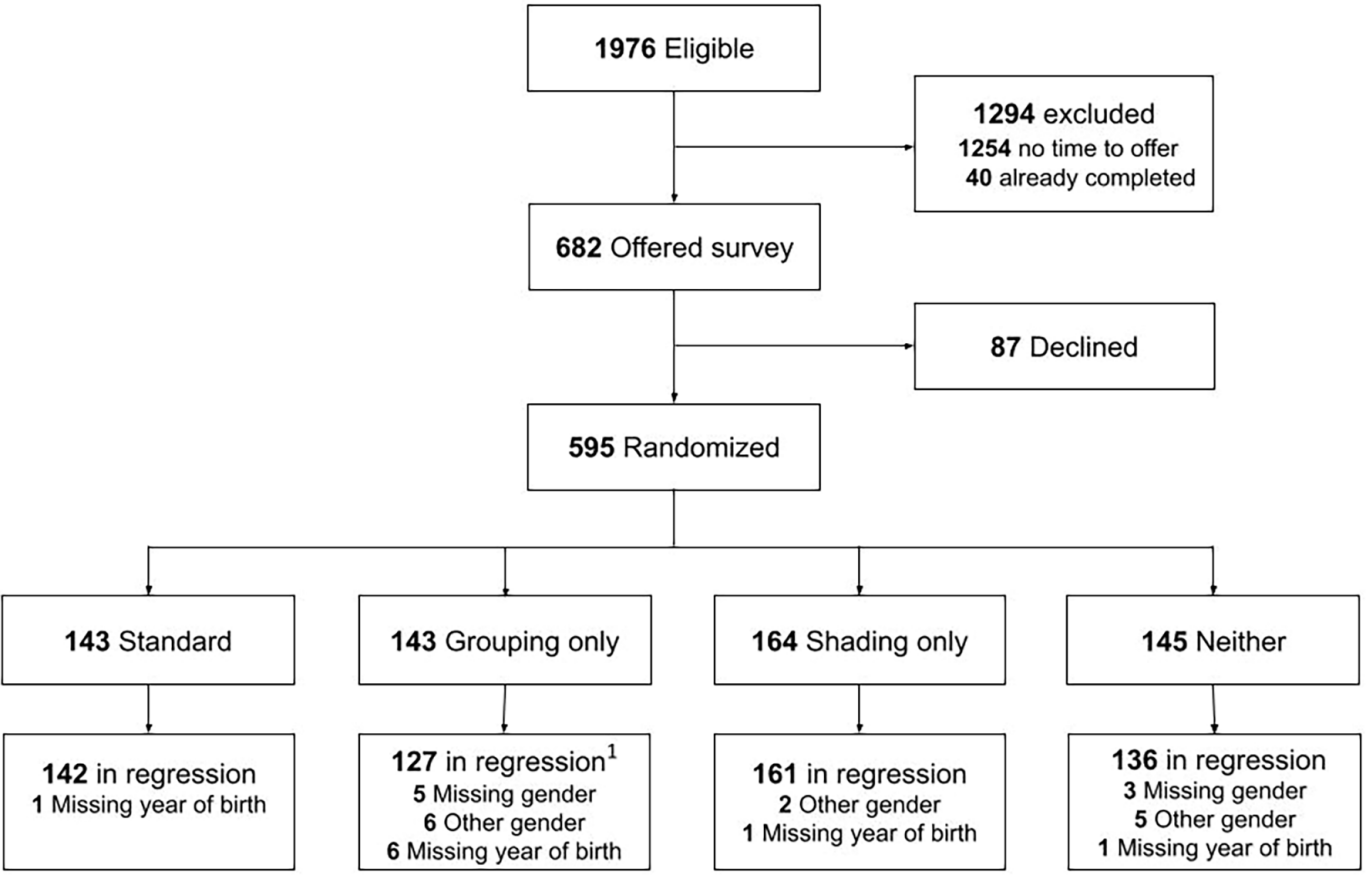
Study flow chart. ^1^One participant was missing year of birth and gender, therefore a total of 16 participants were excluded from the analysis for the grouping only version. ^2^For the scoring methods using both part A and part B of the ASRS, an additional 26 participants that did not complete part B were excluded from the analysis, so the total participants included in the analysis was 540.

**Figure 1** presents the participant flow. Of the 1,976 eligible individuals, 1,254 were not offered the survey due to staff availability, 40 had completed it previously, 87 declined, and 595 completed it. Although age and gender/sex differed significantly between groups, the differences were not practically meaningful: mean age ranged from 40 to 45 years, and the proportion of women ranged from 77% to 78% (**Supplementary Material 3**). There was variation in the percentage of patients offered the survey across days of the week, but this difference was not statistically significant (**Supplementary Material 4**). Additionally, in total 45% of surveys (IQR 27%-57%) were completed in the morning and the remaining in the afternoon.

**Table 2** provides the participant characteristics. No statistically significant differences were found between participants of each survey version. The mean age across groups ranged from 39 to 41 years. Overall, 79% of participants identified as women; 54% identified as White and 33% as Asian. Additionally, 85% reported having completed at least some post-secondary education. Approximately 81% of participants self-reported having a family member diagnosed with ADHD, 14% reported being diagnosed with ADHD and 23% suspected they had ADHD.

**Table 2:** Participant characteristics.

Characteristic	Standard N=143	Grouping only N=143	Shading only N=164	Neither N=145	Total N=595	P-value
Gender						
Woman, n (%)	114 (79.7)	102 (73.9)	132 (80.5)	113 (79.6)	461 (78.5)	0.72 (chi-square)
Man, n (%)	29 (20.3)	30 (21.7)	30 (18.3)	24 (16.9)	113 (19.3)	
Other, n (%)	0 (0.0)	6 (4.3)	2 (1.2)	5 (3.5)	13 (2.2)	
	n = 143	n = 138	n = 164	n = 142	N = 587	
Age* (years)	39.3 (11.7)	38.7 (12.0)	39.1 (11.7)	41.0 (13.6)	39.5 (12.3)	0.64 (ANOVA)
	n = 142	n = 137	n = 163	n = 144	N = 586	
Education						
No high school, n (%)	0 (0.0)	2 (1.4)	2 (1.2)	2 (1.4)	6 (1.0)	0.24 (chi-square)
High school, n (%)	17 (11.9)	20 (14.1)	21 (12.9)	27 (18.8)	85 (14.4)	
Some post-sec, n (%)	87 (60.8)	92 (64.8)	100 (61.4)	95 (66.0)	374 (63.2)	
Master/PhD, n (%)	39 (27.1)	28 (19.7)	40 (24.5)	20 (13.9)	127 (21.5)	
	n = 143	n = 142	n = 163	n = 144	N = 592	
Ethnicity						
Asian, n (%)	49 (34.3)	47 (33.1)	56 (34.4)	41 (28.3)	193 (32.5)	0.63 (chi-square)
Black, n (%)	4 (2.8)	9 (6.3)	6 (3.7)	5 (3.4)	24 (4.0)	
Hispanic, n (%)	4 (2.8)	3 (2.1)	3 (1.8)	2 (1.4)	12 (2.0)	
Indigenous, n (%)	4 (2.8)	5 (3.5)	4 (2.5)	8 (5.5)	21 (3.5)	
Mixed, n (%)	4 (2.8)	2 (1.4)	5 (3.1)	2 (1.4)	13 (2.2)	
Haw or Pac Isle, n (%)	0 (0.0)	0 (0.0)	0 (0.0)	2 (1.4)	2 (0.3)	
White, n (%)	76 (53.1)	76 (53.5)	88 (54.0)	82 (56.6)	322 (54.3)	
Other, n (%)	2 (1.4)	0 (0.0)	1 (0.6)	3 (2.1)	6 (1.0)	
	n = 143	n = 142	n = 163	n = 145	N = 593	
ADHD Family History						
Yes, n (%)	116 (81.1)	119 (83.9)	128 (79.0)	116 (80.0)	479 (80.8)	0.79 (chi-square)
No, n (%)	27 (18.9)	23 (16.2)	35 (21.0)	29 (20.0)	114 (19..2)	
	n = 143	n = 142	n = 163	n = 145	N = 593	
ADHD diagnosed, n (%)	18 (12.6)	23 (16.1)	17 (10.4)	27 (18.6)	85 (14.3)	0.4 (chi-square)
ADHD suspected, n (%)	36 (25.2)	32 (22.4)	40 (24.4)	28 (19.3)	136 (22.9)	
Neither, n (%)	89 (62.2)	88 (61.5)	107 (65.2)	90 (62.1)	374 (62.9)	
	n = 143	n = 143	n = 164	n = 145	N = 595	

*Age reported as mean (Standard Deviation).

Percentages may not equal 100% due to rounding. Total participants may not equal 595 due to missing data.

**Table 3** shows the percentage of screen positives for each version of the ASRS. The screen-positive rate across all participants was 32%, with the highest screening positive rates in participants who had been diagnosed or who suspected they had ADHD. Additionally with diagnosed participants excluded, the screen positive rate was 24% (120/510 participants).

**Table 3:** Percentage and number of positive responses for each survey version based on ADHD status.

Population	Standard^1^	Grouping only^1^	Shading only^1^	Neither^1^	Total^1^
All participants	33.3%, 48 (143)	27.3%, 39 (143)	31.7%, 52 (164)	36.6%, 53 (145)	32.3%, 192 (595)
Diagnosed	88.9%, 16 (18)	87.0%, 20 (23)	88.2%, 15 (17)	77.8%, 21 (27)	84.7%, 72 (85)
Suspected	58.3%, 21 (36)	53.1%, 17 (32)	67.5%, 27 (40)	71.4%, 20 (28)	62.5%, 85 (136)
Not diagnosed or suspected	12.4%, 11 (89)	2.3%, 2 (88)	9.4%, 10 (107)	13.3%, 12 (90)	9.4%, 35 (374)

^1^% positive responses, number of positive responses (total responses).

**Table 4** provides the logistic regression results. Variables associated with the screen positive rate were previous diagnoses of ADHD (OR 47.4, 95% CI 23.1–97.0, p < 0.0001), suspected ADHD (OR 16.2, 95% CI 9.7–27.2, p = 0.0009), and age (OR 0.97, 95% CI 0.96-0.99, p – 0.01). ASRS survey versions were not statistically significant predictors of ADHD positive screening (p ≥ 0.05). The results were similar for the other three other scoring methods except for ‘Part A and B, Dichotomous scoring,’ not shading reduced the odds of screen positive (OR 0.72, 95% CI 0.54–0.96) and for ‘Part A, Scaled scoring,’ not grouping increased the odds of screen positive (1.35, 95% CI 1.03–1.76) (**Supplementary Material 5**).

**Table 4:** Logistic regression of possible factors associated with a positive ADHD screen.

Covariate	N	Odds ratio	95% confidence intervals	P-value (< 0.05)
Grouping				
Yes	269	Ref	Ref	Ref
No	297	1.25	0.98 – 1.58	0.07
Shading				
Yes	303	Ref	Ref	Ref
No	263	0.88	0.69 – 1.12	0.29
Grouping & Shading (interaction)		1.21	0.95 – 1.53	0.12
Gender				
Woman	456	Ref	Ref	Ref
Man	110	0.73	0.38 – 1.38	0.33
ADHD				
Not suspected or diagnosed	357	Ref		
Diagnosed	78	47.4	23.1-97.0	**<0.0001**
Suspected	131	16.2	9.7-27.2	**0.0009**
Age	566	0.97	0.96 – 0.99	0.01

^1^Bold indicates statistical significance.

^2^Excluded ‘other’ from gender because it added complexity without changing the results of the model.

**Table 5** provides the characteristics of participants diagnosed with ADHD. In total, 85 participants who completed the survey self-reported an ADHD diagnosis. Participants were diagnosed at a median age of 27 (IQR 20-35); had been diagnosed for a median of 3 years (IQR 0–8); and over half (52%) were currently taking ADHD medications.

**Table 5:** Characteristics of participants diagnosed with ADHD (n = 85).

Characteristics	
Currently taking ADHD medication (n, %)	44 (52%)
Current age (median years, IQR)^a^	33 (27-42)
Age diagnosed (median years, IQR)^b^	27 (20-35)
Median years since diagnosed (years)^c^	3 (0–8)
Specialty of physician that diagnosed ADHD	
Family physician (n, %)	37 (44%)
Psychologist (n, %)	15 (18%)
Psychiatrist (n, %)	27 (32%)
Don’t know (n, %)	6 (7%)
Age diagnosed^b^	
≤12 years old (n, %)	7 (9%)
13–18 years old (n, %)	10 (13%)
19–25 years old (n, %)	16 (21%)
26-35 years old (n, %)	24 (32%)
>35 years old (n, %)	18 (24%)

a Missing 1 participants who did not provide year of birth.

b Missing 10 participants who did not provide year of birth or year of diagnosis.

c Missing 9 participants who did not provide year of diagnosis. Percentages may not equal 100% due to rounding.

## Discussion

To our knowledge, this is the first study published to examine whether the format of the ASRS increases the likelihood of a positive ADHD screen. Although no shading (grouping only) tended to decrease the positive rate and no grouping (shading only) tended to increase it—with both effects reaching statistical significance in other scoring methods—neither effect was statistically significant when using the standard scoring method. These findings suggest primary care providers should continue using the ASRS version with grouping and shading, especially since it is faster to score.

These results differ from our original hypothesis, which predicted that eliminating shading and grouping would reduce the screen-positive rate. The trend toward increased positive screens not grouping may reflect satisficing behavior, when all 18 items on the ASRS appear as one group, respondents may be more likely to provide quick, adequate answers rather than optimal ones (15). Not shading, meanwhile, showed a non-significant trend toward reducing positive screens. This is consistent with prior research suggesting that color cues are less influential when each response option is clearly labeled, as is the case with the ASRS (16). This may also explain why not shading with dichotomous scoring for Parts A and B was associated with a statistically significant reduction in the odds of a positive screen, it may have made it harder for respondents to associate the header labels with the individual questions further down the ASRS.

The screen-positive rate of the ASRS in this trial was substantially higher than the estimated population prevalence, of 2-7% (2–5). The rate was 32% when all participants were included and 24% when those with a prior ADHD diagnosis were excluded. Although the patient population at Shifa is not fully representative of Canadian adults (55% vs. 70% Caucasian, 79% vs. 50% women, and 85% vs. 58% with post-secondary education, respectively), the results may be reflective of the broader Canadian population as screen positive rate aligns with findings from similar studies conducted in the UK (26%) and the US (17%) (12, 20–22). Further, it is unlikely that the rate is inflated due to selection bias. Even if all 87 patients who declined participation had screened negative, the positive rate would remain approximately 28%. Furthermore, the 1,254 patients who were not offered the survey were excluded due to staff time limitations rather than the likelihood of having ADHD, and the observed differences in gender/sex and age between those offered and not offered the survey were not practically meaningful.

This high screen positive rate highlights the importance of conducting a comprehensive ADHD evaluation in addition to the ASRS. Such an assessment should involve a detailed clinical interview and collateral reports from informants to evaluate symptoms across the lifespan, assess functional impairments, and rule out alternative diagnoses (23).

However, the feasibility of conducting comprehensive assessments within primary care is questionable. First, the time required for a proper evaluation—often estimated at a minimum of two hours—far exceeds the typical 15-minute appointment slot available in Canadian family practice, where providers are already facing significant time constraints (24–26). Second, many primary care providers may lack the necessary expertise or confidence to carry out these assessments (8, 24, 27). According to the recently published Adult ADHD Assessment Quality Assurance Standard, clinicians need formal training in ADHD, supervised experience assessing at least 10–20 ADHD cases, and a minimum of one year’s experience working in a mental health setting to be considered qualified (24).

This places primary care providers in a precarious position. There is increasing demand and pressure to perform ADHD assessments, but they do not have the time, expertise, or tools to properly conduct the assessments (6). Indeed, in Canada, diagnosing and managing ADHD has increasingly shifted to family physicians over the past decade, resulting in more than half of all ADHD assessments now being conducted in primary care, and this trend was also seen in this study (7, 28). This raises the risk of overdiagnosis, which was observed in this trial (18% of patients had a diagnoses of ADHD compared to 2-7% prevalence in the adult population). This can have serious consequences, including inappropriate treatment, medication misuse, adverse drug effects, the psychological burden of an ADHD label, and the misallocation of healthcare resources (29, 30).

It would be useful to have screening tools in primary care that can better distinguish between ADHD and other related conditions and identify malingering, while still being feasible in the busy clinical setting. One potential approach is to adapt existing tools for this purpose. In the case of the ASRS, modifications such as removing shading for positive questions, not grouping Part A and Part B questions, and using different scoring methods did not appear to improve the effectiveness. However, other strategies may prove beneficial—such as revising the scoring criteria, raising the threshold for a positive result, incorporating questions to screen for comorbid conditions and malingering, or combining the ASRS with other tools that address these areas. For instance, the ADHD Symptom Infrequency Scale (ASIS) includes embedded items designed to detect malingering (18).

This study has several limitations. Although we obtained some demographic and diagnostic data from individuals who self-reported an ADHD diagnosis (**Table 5**), we do not have sufficient information to assess the accuracy of these diagnoses. We had attempted to gather additional details on diagnostic methods through a question asking participants how they were diagnosed (e.g., clinical interview, screening tool, or cognitive assessment); however, participants may not have accurately understood the terminology, so we have not reported this data. Lastly, our findings may not generalize to rural populations, as planned recruitment from two rural clinics was not completed due to staffing changes.

## Conclusions

Shading screen-positive responses and grouping the key questions in Part A does not appear to impact the screen positive rate. However, the high observed screen-positive rate is a concern in the context of limited time and expertise of primary care providers. Additional screening tools would be useful to help primary care providers better identify patients who have ADHD.

## Data Availability

The raw data supporting the conclusions of this article will be made available by the authors, without undue reservation.
