# Supplementary material for: Does the format of the adult ADHD self-report scale influence screen-positive rates? A randomized controlled trial in primary care
Source: Front Psychiatry. 2026 Jan 2;16:1646293. doi: 10.3389/fpsyt.2025.1646293 (PMC12808415; doi:10.3389/fpsyt.2025.1646293)
Supplement: Supplementary file 1 [file DataSheet1.pdf]

Supplementary Material

1 Demographic questions on survey

A - Demographic questions on survey

Year of Birth: ______

---

1. Gender Identity:

   Mark only one

- Man
- Woman
- Non-binary
- Transgender
- Prefer not to answer
- Other: _______

---

2. Level of Education:

   Mark only one

- High school diploma, or equivalent
- Post-secondary (ex. university, college), some or completed
- Professional/Graduate degree (ex. MA, MSc, PhD), some or completed
- Other: _______

---

3. Ethnicity (if two or more please list in other):

   Mark only one

- White
- Black
- Asian
- Hispanic
- Indigenous
- Native Hawaiian or Pacific Islander
- Other: ______

---

4. Do you have a parent, sibling, or child that has been diagnosed with ADHD?

   Mark only one

- Yes
- No
- Other: ______

---

5. Have you been diagnosed with ADHD?

   Mark only one

- Yes *Skip to questions 5a - 5d*
- No *Skip to question 5e*
- Other: ______ *Skip to question 5e*

5a. Who diagnosed you with ADHD?

Mark only one

- Family Physician
- Psychiatrist
- Psychologist
- Other: ______

5b. In which year were you diagnosed with ADHD? ______

5c. What methods were used to diagnose for ADHD? (Check all that apply)

- Interview
- Cognitive Testing
- ADHD Screening form
- Other: ______

5d. Are you currently taking your ADHD medication regularly (more than 3 days/week in the past 2 weeks)?

Mark only one

- Yes
- No
- Other:

5e. Do you suspect you may have ADHD?

Mark only one

- Very unlikely
- Unlikely
- Likely
- Very likely

2 Versions of survey

Standard (grouping and shading)

Please answer the questions below, rating yourself on each of the criteria shown using the scale on the right side of the page. Please answer all questions, and choose the response that best describes how you have felt and conducted yourself over the past 6 months.

Questions	Never	Rarely	Sometimes	Often	Very Often
How often do you have trouble wrapping up the final details of a project, once the challenging parts have been done?					
How often do you have difficulty getting things in order when you have to do a task that requires organization?					
How often do you have problems remembering appointments or obligations?					
When you have a task that requires a lot of thought, how often do you avoid or delay getting started?					
How often do you fidget or squirm with your hands or feet when you have to sit down for a long time?					
How often do you feel overly active and compelled to do things, like you were driven by a motor?					
Part A					
How often do you make careless mistakes when you have to work on a boring or difficult project?					
How often do you have difficulty keeping your attention when you are doing boring or repetitive work?					
How often do you have difficulty concentrating on what people say to you, even when they are speaking to you directly?					
How often do you misplace or have difficulty finding things at home or at work?					
How often are you distracted by activity or noise around you?					
How often do you leave your seat in meetings or other situations in which you are expected to remain seated?					
How often do you feel restless or fidgety?					
How often do you have difficulty unwinding and relaxing when you have time to yourself?					
How often do you find yourself talking too much when you are in social situations?					
When you're in a conversation, how often do you find yourself finishing the sentences of the people you are talking to, before they can finish them themselves?					
How often do you have difficulty waiting your turn in situations when turn taking is required?					
How often do you interrupt others when they are busy?					
Part B					

Grouping only

Please answer the questions below, rating yourself on each of the criteria shown using the scale on the right side of the page. Please answer all questions, and choose the response that best describes how you have felt and conducted yourself over the past 6 months.

Questions	Never	Rarely	Sometimes	Often	Very Often
How often do you have trouble wrapping up the final details of a project, once the challenging parts have been done?					
How often do you have difficulty getting things in order when you have to do a task that requires organization?					
How often do you have problems remembering appointments or obligations?					
When you have a task that requires a lot of thought, how often do you avoid or delay getting started?					
How often do you fidget or squirm with your hands or feet when you have to sit down for a long time?					
How often do you feel overly active and compelled to do things, like you were driven by a motor?					
Part A					
How often do you make careless mistakes when you have to work on a boring or difficult project?					
How often do you have difficulty keeping your attention when you are doing boring or repetitive work?					
How often do you have difficulty concentrating on what people say to you, even when they are speaking to you directly?					
How often do you misplace or have difficulty finding things at home or at work?					
How often are you distracted by activity or noise around you?					
How often do you leave your seat in meetings or other situations in which you are expected to remain seated?					
How often do you feel restless or fidgety?					
How often do you have difficulty unwinding and relaxing when you have time to yourself?					
How often do you find yourself talking too much when you are in social situations?					
When you're in a conversation, how often do you find yourself finishing the sentences of the people you are talking to, before they can finish them themselves?					
How often do you have difficulty waiting your turn in situations when turn taking is required?					
How often do you interrupt others when they are busy?					
Part B					

Shading only

Please answer the questions below, rating yourself on each of the criteria shown using the scale on the right side of the page. Please answer all questions, and choose the response that best describes how you have felt and conducted yourself over the past 6 months.

Questions	Never	Rarely	Sometimes	Often	Very Often
How often do you have trouble wrapping up the final details of a project, once the challenging parts have been done?					
How often do you have difficulty getting things in order when you have to do a task that requires organization?					
How often do you have problems remembering appointments or obligations?					
When you have a task that requires a lot of thought, how often do you avoid or delay getting started?					
How often do you fidget or squirm with your hands or feet when you have to sit down for a long time?					
How often do you feel overly active and compelled to do things, like you were driven by a motor?					
How often do you make careless mistakes when you have to work on a boring or difficult project?					
How often do you have difficulty keeping your attention when you are doing boring or repetitive work?					
How often do you have difficulty concentrating on what people say to you, even when they are speaking to you directly?					
How often do you misplace or have difficulty finding things at home or at work?					
How often are you distracted by activity or noise around you?					
How often do you leave your seat in meetings or other situations in which you are expected to remain seated?					
How often do you feel restless or fidgety?					
How often do you have difficulty unwinding and relaxing when you have time to yourself?					
How often do you find yourself talking too much when you are in social situations?					
When you're in a conversation, how often do you find yourself finishing the sentences of the people you are talking to, before they can finish them themselves?					
How often do you have difficulty waiting your turn in situations when turn taking is required?					
How often do you interrupt others when they are busy?					

Neither

Please answer the questions below, rating yourself on each of the criteria shown using the scale on the right side of the page. Please answer all questions, and choose the response that best describes how you ha felt and conducted yourself over the past 6 months.

Questions	Never	Rarely	Sometimes	Often	Very Often
How often do you have trouble wrapping up the final details of a project, once the challenging parts have been done?					
How often do you have difficulty getting things in order when you have to do a task that requires organization?					
How often do you have problems remembering appointments or obligations?					
When you have a task that requires a lot of thought, how often do you avoid or delay getting started?					
How often do you fidget or squirm with your hands or feet when you have to sit down for a long time?					
How often do you feel overly active and compelled to do things, like you were driven by a motor?					
How often do you make careless mistakes when you have to work on a boring or difficult project?					
How often do you have difficulty keeping your attention when you are doing boring or repetitive work?					
How often do you have difficulty concentrating on what people say to you, even when they are speaking to you directly?					
How often do you misplace or have difficulty finding things at home or at work?					
How often are you distracted by activity or noise around you?					
How often do you leave your seat in meetings or other situations in which you are expected to remain seated?					
How often do you feel restless or fidgety?					
How often do you have difficulty unwinding and relaxing when you have time to yourself?					
How often do you find yourself talking too much when you are in social situations?					
When you're in a conversation, how often do you find yourself finishing the sentences of the people you are talking to, before they can finish them themselves?					
How often do you have difficulty waiting your turn in situations when turn taking is required?					
How often do you interrupt others when they are busy?					

3 Comparison of participants that completed, declined, and were not offered the survey

Characteristic	Completed (n=595)	Declined (n=87)	Not offered (n=1294)	p-value
Woman	461 (77.5%)	68 (78.2%)	994 (76.8%)	0.01 Fisher's exact test
Man	113 (19.0%)	19 (21.8%)	296 (22.9%)	
Other	13 (2.2%)	0 (0.0%)	2 (0.2%)	
Unknown	8 (1.3%)	0 (0.0%)	2 (0.2%)	
Age (mean, SD)	39.5 (12.3); n=586	44.7 (11.4); n=87	40.7 (12.7); n=1292	0.0007 (ANOVA)

Gender is reported for participants that completed the survey and sex is reported from participants that declined and were not offered.

4 Percentage of patients offered the survey by day of the week

	Monday	Tuesday	Wednesday	Thursday	Friday	Saturday
Number of days	9	10	10	9	9	7
Median	47.8%	51.7%	34.9%	45.6%	57.1%	62.5%
25th percentile	22.3%	35.5%	14.2%	27.0%	37.9%	17.0%
75th percentile	66.5%	59.3%	60.4%	59.9%	73.0%	75.0%

Kruskal-Wallis Test conducted, percentages among days were not statistically significant, p=0.560.
The total possible days for distribution of the form was 10 days for Monday, Tuesday and Wednesday and 9 days for Thursday, Friday, Saturday. Shifa Medical Clinic was closed on one of the Mondays during the study period and two of the Saturdays, so the form was only distributed on 9 Mondays and 7 Saturdays. The clinic was closed on Sundays.

5 Analysis using other scoring methods

5.1 Part A and B, Dichotomous scoring (ie, each question is scored as either positive or negative)

Cutoff ≥ 9 out of 18 for a positive screen.

Percentage and number of positive responses for each form version based on ADHD status

Population	Standard[1]	Grouping only[1]	Shading only[1]	Neither[1]	Total[1]
All participants	36.0%, 50 (139)	33.3%, 45 (136)	38.3%, 59 (154)	37.1%, 52 (140)	36.3%, 206 (569)
Diagnosed	100.0%, 18 (18)	91.3%, 21 (23)	100.0%, 17 (17)	88.9%, 24 (27)	94.0%, 80 (85)
Suspected	71.4%, 25 (35)	65.6%, 21 (32)	79.5%, 31 (39)	80.8%, 21 (26)	74.2%, 98 (132)
Not diagnosed or suspected	8.1%, 7 (86)	3.7%, 3 (81)	11.2%, 11 (98)	8.1%, 7 (87)	8.0%, 28 (352)

[1]% positive responses, number of positive responses (total responses)

Logistic regression of factors that may be associated with a positive ADHD screen

Variable	N	OR	95% Confidence Intervals	p-value
Grouping				
Yes	258	Ref		Ref
No	282	1.29	0.97 - 1.73	0.08
Shading				
Yes	289	Ref		Ref
**No**	251	0.72	0.54 – 0.96	**0.02**
Grouping/Shading (interaction)		1.06	0.80 - 1.42	0.67
Gender				
Woman	433	Ref		Ref
Man	107	0.57	0.26 – 1.22	0.15
ADHD				
Not suspected or diagnosed	335	Ref		Ref
Diagnosed	78	220.4	77.0 – 631.0	**< 0.0001**
Suspected	127	42.4	22.9 – 78.6	**0.002**
Age	540	0.97	0.94 - 0.99	**0.006**

Bold indicates statistical significance.
Excluded ‘other’ from gender because it did not change the model.

5.2 Part A, Scaled scoring (ie, 0 being never and 4 being very often)

Cutoff ≥ 14 out of 24 for a positive screen.

Percentage of positive responses for each form version based on ADHD status

Population	Standard[1]	Grouping only[1]	Shading only[1]	Neither[1]	Total[1]
All participants	30.8%, 44 (143)	27.3%, 39 (143)	30.5%, 50 (164)	35.2%, 51 (145)	30.9%, 184 (595)
Diagnosed	88.9%, 16 (18)	87.0%, 20 (23)	88.2%, 15 (17)	88.9%, 24 (27)	88.2%, 75 (85)
Suspected	55.6%, 20 (36)	56.3%, 18 (32)	67.5%, 27 (40)	75.0%, 21 (28)	63.2%, 86 (136)
Not diagnosed or suspected	8.0%, 8 (89)	1.1%, 1 (88)	7.5%, 8 (107)	6.7%, 6 (90)	6.1%, 23 (374)

[1]% positive responses, number of positive responses (total responses)

Logistic regression of factors that may be associated with a positive ADHD screen

Variable	N	OR	95% Confidence Intervals	p-value
Grouping				
Yes	269	Ref		Ref
No	297	1.35	1.03-1.76	**0.03**
Shading				
Yes	303	Ref		Ref
No	263	0.89	0.68 – 1.16	0.38
Grouping/Shading (interaction)		1.22	0.94 – 1.59	0.14
Gender				
Woman	456	Ref		Ref
Man	110	0.94	0.47 – 1.91	0.87
ADHD				
Not suspected or diagnosed	357	Ref		Ref
Diagnosed	78	107.3	47.2 – 244.3	**< 0.0001**
Suspected	131	29.1	16.3 – 52.0	**0.0002**
Age	566	0.97	0.95 - 0.99	**0.01**

Bold indicates statistical significance.
Excluded 'other' from gender because it did not change the model.

5.3 Part A and B, Scaled scoring (ie, 0 being never and 4 being very often)

Cutoff ≥ 35 out of 72 for a positive screen.

Percentage and number of positive responses for each form version based on ADHD status

Population	Standard[1]	Grouping only[1]	Shading only[1]	Neither[1]	Total[1]
All participants	40.3%, 56 (139)	37.0%, 50 (136)	40.9%, 63 (154)	44.3%, 62 (140)	40.7%, 231 (569)
Diagnosed	100.0%, 18 (18)	91.3%, 21 (23)	100.0%, 17 (17)	88.9%, 24 (27)	94.1%, 80 (85)
Suspected	82.9%, 29 (35)	78.1%, 25 (32)	84.6%, 33 (39)	84.6%, 22 (26)	82.6%, 109 (132)
Not diagnosed or suspected	10.5%, 9 (86)	4.9%, 4 (81)	13.3%, 13 (98)	18.4%, 16 (87)	12.0%, 42 (352)

[1]% positive responses, number of positive responses (total responses)

Logistic regression of factors that may be associated with a positive ADHD screen

Variable	N	OR	95% Confidence Intervals	p-value
Grouping				
Yes	258	Ref		Ref
No	282	1.27	0.97 - 1.67	0.08
Shading				
Yes	289	Ref		Ref
No	251	0.89	0.69 – 1.17	0.41
Grouping/Shading (interaction)		1.19	0.91 - 1.56	0.20
Gender				
Woman	433	Ref		Ref
Man	107	1.377	0.69 – 2.75	0.36
ADHD				
Not suspected or diagnosed	335	Ref		Ref
Diagnosed	78	111.2	41.2 – 300.3	**< 0.0001**
Suspected	127	38.4	21.2 – 69.5	**0.0002**
Age	540	0.97	0.95 - 0.99	**0.008**

Bold indicates statistical significance.
Excluded 'other' from gender because it did not change the model.
